# Evaluating Molecular Mechanical Potentials for Helical Peptides and Proteins

**DOI:** 10.1371/journal.pone.0010056

**Published:** 2010-04-07

**Authors:** Erik J. Thompson, Allison J. DePaul, Sarav S. Patel, Eric J. Sorin

**Affiliations:** 1 Department of Chemical Engineering, California State University Long Beach, Long Beach, California, United States of America; 2 Department of Chemistry & Biochemistry, California State University Long Beach, Long Beach, California, United States of America; German Cancer Research Center, Germany

## Abstract

Multiple variants of the AMBER all-atom force field were quantitatively evaluated with respect to their ability to accurately characterize helix-coil equilibria in explicit solvent simulations. Using a global distributed computing network, absolute conformational convergence was achieved for large ensembles of the capped A_21_ and F_s_ helical peptides. Further assessment of these AMBER variants was conducted via simulations of a flexible 164-residue five-helix-bundle protein, apolipophorin-III, on the 100 ns timescale. Of the contemporary potentials that had not been assessed previously, the AMBER-99SB force field showed significant helix-destabilizing tendencies, with beta bridge formation occurring in helical peptides, and unfolding of apolipophorin-III occurring on the tens of nanoseconds timescale. The AMBER-03 force field, while showing adequate helical propensities for both peptides and stabilizing apolipophorin-III, (i) predicts an unexpected decrease in helicity with ALA→ARG^+^ substitution, (ii) lacks experimentally observed 3_10_ helical content, and (iii) deviates strongly from average apolipophorin-III NMR structural properties. As is observed for AMBER-99SB, AMBER-03 significantly overweighs the contribution of extended and polyproline backbone configurations to the conformational equilibrium. In contrast, the AMBER-99φ force field, which was previously shown to best reproduce experimental measurements of the helix-coil transition in model helical peptides, adequately stabilizes apolipophorin-III and yields both an average gyration radius and polar solvent exposed surface area that are in excellent agreement with the NMR ensemble.

## Introduction

Simulating protein dynamics remains a daunting task in computational chemistry and biophysics. While many advances have been made in the last decade, processor speeds limit the timescales on which biomolecules can be simulated using explicit representations of aqueous solvent to the sub-microsecond timescale–far below the threshold of most interesting biomolecular events. More importantly, the ability of contemporary molecular models to accurately characterize the energetics of biopolymers depends greatly on the computational methods used to assess the models in question and the systems studied during such assessment. It has been shown that very subtle modifications to commonly used molecular mechanical potentials, such as changes to the scaling factors applied to non-bonded interactions [1], can significantly alter the behavior of those potentials with respect to stabilizing, or destabilizing, protein substructure. Most often, these modifications have aimed to improve upon the torsional potentials around the φ and ψ dihedrals in protein backbones [2], which were fit to the relative quantum mechanical energies of alternate rotamers of small GLY and ALA peptides in Cornell's seminal AMBER-94 force field [3]: (a) AMBER-96 recalibrates these parameters to accurately predict energy differences between constrained and extended α-helical conformations of ALA peptides [4]; (b) AMBER-99 refits the parameterization using calculations on representative ALA tetrapeptides [5]; and (c) the Garcia-Sanbonmatsu variant of AMBER-94 (commonly referred to as AMBER-GS) zeroes both torsional potentials [6].

In an effort to properly assess the conformational preferences and equilibria of model systems simulated under the various force fields in use by the computational community, we thus set out to systematically study contemporary potentials in their application to one of the most ubiquitous and fundamental of protein substructures: the α-helix [1], [7], [8], [9]. These studies included the simulation of large ensembles of model helical peptides starting from fully helical and fully unfolded states to convergence of conformational equilibrium on the hundreds of nanoseconds timescale. Our results allowed absolute characterization of the equilibrium helical content and dynamics of many published molecular models including the AMBER-94, AMBER-96, AMBER-99, and AMBER-GS force fields. At that time, a new variant dubbed AMBER-99φ, which replaces the φ potential in the *helix-destabilizing* AMBER-99 with that of the *helix-friendly* AMBER-94 force field, was shown to yield the best agreement with numerous experimental characterizations of the helical F_s_ peptide including the helix folding time, mean 3_10_ helicity, mean residue dwell time in the coil state, mean radius of gyration, and Lifson-Roig (LR) parameters. AMBER-99φ also gave the best agreement with quantum mechanical sampling of the alanine dimer and a survey of alanine conformations in the Protein Data Bank (PDB) [7].

**Figure 1:**
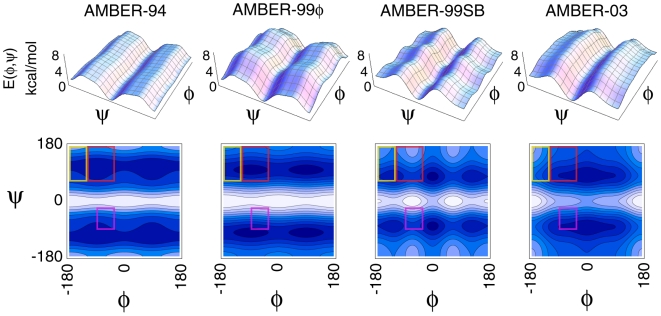
The φ/ψ potentials for the four AMBER force fields compared in this work. Landscapes are shown to highlight relative magnitude differences between the potentials (top), while contours more clearly display the positions of local and global minima and maxima for each force field. The regions corresponding to alpha helix (purple), beta strand (yellow), and polyproline type II (red) conformations are indicated based on the definitions used by Garcia for Ala peptides [11].

In recent years, Duan and coworkers have published their third generation AMBER-03 force field [10], which fits the dihedral potentials to new quantum mechanical calculations using a low-dielectric continuum model, and Simmerling and coworkers have reported on their AMBER-99 variant, commonly referred to as AMBER-99SB, which includes reparameterized φ/ψ backbone torsions that were fit to *ab initio* calculations of ALA and GLY tetrapeptides [2]. The backbone torsional potentials for each of these force fields, as well as the previously characterized AMBER-94 and AMBER-99φ force fields, are shown in Figure 1, which highlights both the relative magnitude of the respective φ/ψ potentials and the locations of energetic minima and maxima. Regions of the φ/ψ space corresponding to alpha helix, beta strand, and polyproline backbone configurations are also indicated based on the definitions of Garcia for polyalanine peptides [11].

In tandem with non-bonded interactions, these torsional potentials comprise one of the most dominant factors in determining the configurational preferences of each molecular mechanical force field. This article therefore reports on our continued and broadened assessment of contemporary potentials in simulating helical peptides and proteins, which includes evaluation of the conformational equilibrium of large ensembles of small alanine-based peptides, and examines the ability of these force fields to stabilize the native structure of a large and flexible five-helix-bundle lipid-transport protein, apolipophorin-III (apoLp-III) [12], on the 100 ns timescale.

## Methods

### Helical peptide simulations

The capped A_21_ (Ace-A_21_-NMe) and F_s_ (Ace-A_5_[AAAR^+^A]_3_A-NMe) peptides were simulated using the AMBER-99φ [7], AMBER-99SB [2], and AMBER-03 [10] all-atom potentials, which were ported into the GROMACS molecular dynamics (MD) suite [13] as part of our ffAMBER distribution of force field ports (available at http://chemistry.csulb.edu/ffamber/), and quantitatively validated against the AMBER 8 software. The GROMACS suite, known to be one of the fastest MD packages available for biomolecular simulation, was modified for the Folding@Home [14] infrastructure (http://folding.stanford.edu). Following our previous methodology [1], [7], [15], a canonical helix (φ = −57°, ψ = −47°) and a random coil configuration with no helical content were generated and centered in 40 Å cubic boxes. For F_s_ peptide simulations, electroneutrality was achieved by placing three Cl^−^ ions randomly around the solute with minimum ion-ion and ion-solute separations of 5 Å. As the degree of helicity of the F_s_ peptide has been shown to be nearly constant between pH 1 and pH 8 for NaCl concentrations up to 4 M [16], [17], ionic strength should not influence secondary structure in our simulations. The helix and coil conformations were then solvated with 2,075 and 2,065 TIP3P water molecules [18], respectively. After energy minimization using a steepest descent algorithm and solvent annealing for 500 ps of MD with the peptide held fixed, each of these conformations served as the starting point for 1,000 independent MD trajectories in each AMBER potential listed above, which were simulated on ∼10,000 CPUs within the Folding@Home supercluster.

All simulations reported herein were conducted under constant NPT conditions [19] at 1 atm and 305 K, the approximate F_s_ midpoint temperature detected by circular dichroism [20] and ultraviolet resonance Raman [21]. As Ewald approaches are known to overstabilize helices in periodic cells [22], long range electrostatic interactions were treated using the reaction field method [6], [7], [23] with a dielectric constant of 80, and 9 Å cutoffs imposed on all non-bonded interactions, which has proven highly successful in our previous studies of helical peptides [1], [7], [8], [15], [24]. Nonbonded pair lists were updated every 10 steps, and covalent bonds involving hydrogen atoms were constrained with the LINCS algorithm [25]. An integration step size of 2 fs was used, with coordinates stored every 100 ps. As shown in Table 1, the cumulative sampling achieved for the F_s_ peptide totals over 350 µs, with equilibrium sampling of over 150 µs–orders of magnitude longer than the ∼16 ns folding time of F_s_. Similar sampling of the A_21_ peptide was also collected.

**Table 1:** Simulated ensemble statistics for the F_s_ and A_21_ peptides.

		F_s_			A_21_		
Force Field	State*	Max (ns)	Total time (µs)	>EQ (µs)	Max (ns)	Total time (µs)	>EQ (µs)
AMBER-99φ	H	165	70.2	31.4	200	105.2	66.5
	C	170	71.5	32.5	200	108.5	68.7
AMBER-99SB	H	150	40.3	9.9	140	37.8	8.2
	C	130	39.6	9.0	145	39.7	9.2
AMBER-03	H	200	72.8	35.6	200	76.9	38.8
	C	200	73.8	36.2	200	76.2	38.2
**Total**			**368.2**	**154.6**		**444.3**	**229.6**

*Each force field was sampled using 1,000 trajectories starting in the fully helical state (H) and 1,000 trajectories starting in the random coil state (C) with no structured residues.

Max (longest individual trajectory), Total time (total ensemble simulation time), and >EQ (total equilibrium simulation time) are shown for each data set.

### Apolipoprotein simulations

The 164-residue apolipophorin-III protein (PDB 1LS4, model 1) [26] was simulated according to the methods outlined above with minimal changes. Electroneutrality was achieved by placing four Cl^−^ ions and twelve Na^+^ ions randomly around the solute with minimum ion-ion and ion-solute separations of 5 Å, and the temperature was set at 300 K. The simulation box size was set at 66 Å×67 Å×88 Å, and the system was solvated with 12,552 TIP3P water molecules. Simulations were then run in parallel on a local computing cluster using the AMBER-94, AMBER-99φ, AMBER-99SB, and AMBER-03 force fields with 12 Å cutoffs imposed on non-bonded interactions to accurately account for tertiary contacts within the helical bundle. Eight 100 ns simulations per force field were conducted, yielding a total apoLp-III sampling time of 3.2 µs. Each 100 ns simulation of this system, composed of 40,118 atoms in all, required approximately 6 months of wall-clock time on a single 2.5 GHz Xeon processing core.

### Analysis

For the helical peptides and proteins studied herein, structural content per residue was assessed using the Dictionary of Secondary Structure in Proteins (DSSP) [27], which has gained general acceptance among the biophysical community. As in our previous analyses employing DSSP [7], total helical content (H) includes α, 3_10_, and π helical types. Beta structure (B) has been defined as consisting of both β-sheet and β-bridge conformations, and turn regions (T) are distinguished from random coil (C) configurations. In addition, we have assigned polyproline type II (P) structure within our data based on backbone torsions according to the region of the Ramachandran map defined by φ = −75°±50° and ψ = 150°±50°. While seemingly broad, this window was determined via quantitative sampling of the native collagen triple-helix sequences (POG)_10_ and (PPG)_10_ in various AMBER force fields.

Other structural metrics used for comparison of these force fields include the all-atom root-mean-squared-deviation (RMSD) and gyration radius (R_g_), which were calculated using GROMACS analysis tools, as well as the solvent accessible surface area (SASA). We note that analysis tools within molecular simulation packages that calculate SASA, such as the g_sas module in GROMACS, often base definitions of polar and non-polar surface areas on atomic partial charges taken from the molecular potential being used. To avoid this force field dependence due to significant differences in point charges between AMBER variants, and obtain SASA quantities that were based solely on sequence and structure, reported SASA values were calculated using the VEGA package [28], with a probe sphere radius of 1.4 Å and a point density of 24 points/Å^2^. Reported SASA values, including polar and non-polar components, are thus directly comparable between force fields and with the 21 energy-minimized NMR models of apoLp-III [26].

## Results and Discussion

### F_s_ and A_21_ helical peptides

**Figure 2:**
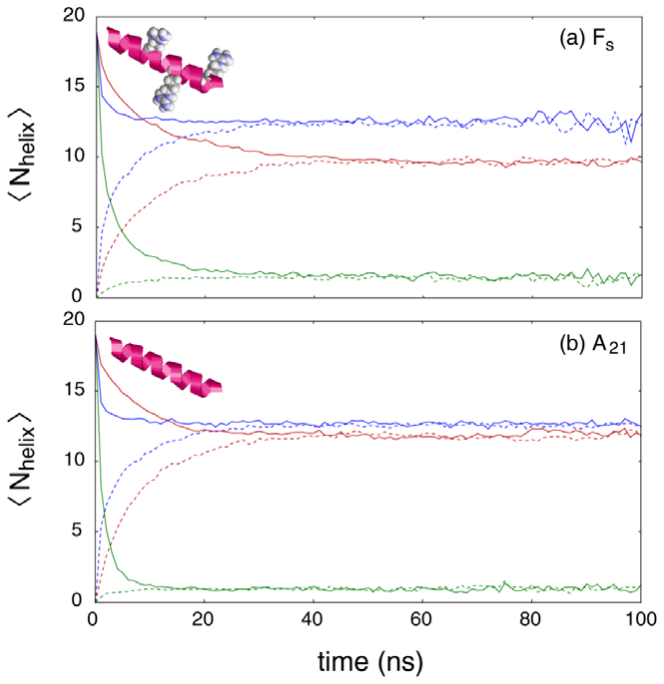
Convergence of mean helical content for the (a) F_s_ and (b) A_21_ ensembles. The AMBER-03 (red), AMBER-99SB (green), and AMBER-99φ (blue) potentials are shown, where <N_helix_> represents the number of helical residues averaged across all runs in a given ensemble of 1,000 simulations. Dotted and solid lines represent simulation ensembles initiated from the fully random coil and fully helical states, respectively. Other structural properties listed in Table 2 show similar convergence. Noise near the 100 ns regime is the result of a limited number of simulations reaching those times following the ensemble convergence that occurs prior to the 40 ns timepoint.

Ensemble convergence of mean helical structure on the 40 ns timescale when using the AMBER-99φ, AMBER-99SB, and AMBER-03 force fields, as demonstrated in Figure 2, is in agreement with our previous observations [1], [7]. Additional structural metrics, all of which showed fully convergent behavior on this timescale, are reported in Table 2. Helical metrics include the mean total helicity (N_helix_), mean 3_10_ helicity (N_310_), mean number of helical segments (N_seg_), and mean longest contiguous helical segment (N_cont_). As our previous efforts have demonstrated that the Lifson-Roig two-state helix-coil model [29] does not adequately capture the complex character of helix-coil equilibria, and LR counting is not an intuitive, nor necessarily accurate, method of assigning helical status to residues in a given conformation, the values shown in Figure 2 and Table 2 come from the generally-accepted DSSP.

**Table 2:** Ensemble averaged equilibrium structural properties for the F_s_ and A_21_ peptides.

Force Field		RMSD (Å)	R_g_ (Å)	N_helix_	N_310_	N_seg_	N_cont_
AMBER-99φ	F_s_	5.3±2.4	9.2±1.3	12.5±3.7	3.4±3.3	1.8±0.6	9.4±4.3
	A_21_	5.1±2.6	9.0±1.5	12.6±3.6	3.3±3.4	1.8±0.6	9.5±4.3
AMBER-99SB	F_s_	7.9±1.5	10.2±2.2	1.5±2.3	1.0±1.7	0.4±0.6	1.3±1.9
	A_21_	8.1±1.6	10.3±2.5	0.9±1.8	0.7±1.4	0.3±0.5	0.9±1.6
AMBER-03	F_s_	6.2±1.9	9.0±1.5	9.6±4.4	1.1±1.9	1.4±0.6	7.8±4.1
	A_21_	5.5±2.6	8.8±1.6	11.9±4.6	0.8±1.6	1.4±0.6	9.9±4.7

RMSD (all-atom root-mean-square deviation), R_g_ (radius of gyration), N_helix_ (number of α-helical residues), N_310_ (number of 3_10_-helical residues), N_seg_ (number of helical segments), and N_cont_ (length of helical segments).

Most noticeable in both Figure 2 and Table 2 is the lack of any significant helical content for both the F_s_ and A_21_ peptides under the AMBER-99SB potential at equilibrium. Additionally, the ensemble average RMSD from the ideal helix is significantly larger than predicted by the AMBER-99φ and AMBER-03 potentials. We note that for these small helical peptides the loss of helicity takes 10–20 ns, and simulations of larger proteins, particularly those that include tertiary stabilization of helical regions, should thus reach significantly longer timescales than this to adequately evaluate force field behavior. Moreover, our AMBER-99SB ensembles showed a slight tendency (∼4% of residues in our equilibrium data set) to form beta structure, composed primarily of bridges of one to two β residues, which was not seen in the AMBER-03 or AMBER-99φ data. Our simulations of polyalanine-based helical peptides thus suggest that this force field not only destabilizes helical structure, as does the original AMBER-99 force field [7], but also exhibits β−structure forming tendencies.

Unlike AMBER-99SB, the AMBER-03 force field of Duan *et al*. clearly exhibits significant helical content in a manner that is qualitatively similar to our AMBER-99φ potential, and a quantitative comparison of these models must therefore focus on the fine details of the established conformational equilibria. Notably, while the AMBER-99φ ensembles converge to nearly identical equilibria for the F_s_ and A_21_ peptides, this is not the case for the AMBER-03 potential, which predicts a significant increase in overall helicity of ∼30% when moving from the ARG^+^ substituted F_s_ peptide to the polyalanine 21-mer. In tandem with this increase in overall helicity, the AMBER-03 potential predicts an increase in average contiguous helix length of ∼20%.

It is well known that polyalanine peptides are insoluble in aqueous solution, which led the Baldwin laboratory to develop many model helical peptides by substituting a limited number of ALA residues with polar amino acids [30], such as the ARG^+^ substitutions made in F_s_. It has been postulated that these sidechains serve not only to make these peptides soluble, but also to stabilize the helical structure within substituted polyalanine peptides [6], [31]. Herein lies an important distinction between these force fields: while AMBER-99φ predicts essentially identical helical propensities between the insoluble polyalanine peptide and its soluble ARG^+^ substituted analog, the AMBER-03 potential predicts *increased* helicity for the insoluble peptide in comparison to its soluble ARG^+^ substituted analog, suggesting that the polar ARG^+^ sidechains actually serve to *destabilize* helical structure in the AMBER-03 potential. While it is difficult to compare this aspect of these potentials when applied to an insoluble peptide, which is a purely non-physical model for which no experimental data can be obtained, the resulting decrease in helicity that accompanies ALA→ARG^+^ substitutions when using AMBER-03 seems to diverge significantly from current knowledge of substituted polyalanine peptides.

**Figure 3:**
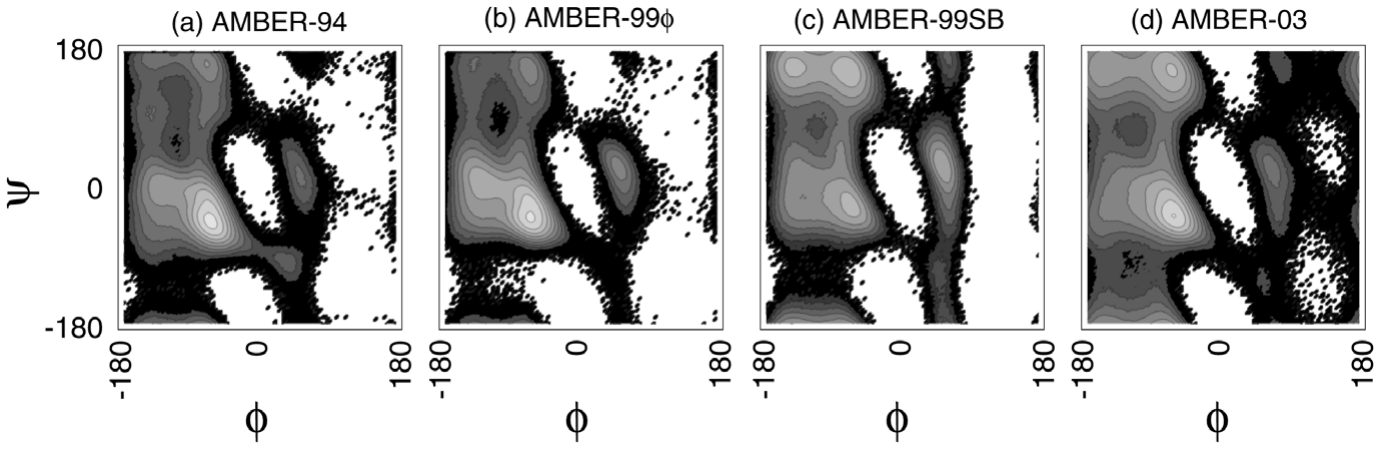
Free energy landscapes projected onto the Ramachandran map. These maps represent equilibrium sampling of the F_s_ peptide in the AMBER force fields evaluated, which have been ordered to match Figure 1 in (a) through (d). Each map consists of backbone torsional values binned in 3° intervals for all residues, and contours represent *k*T units at 305 K, the midpoint temperature of the helical peptide.

We can better understand the differences between these force fields by considering the equilibrium sampling of F_s_ peptide backbone torsions and the resulting Ramachandran maps shown in Figure 3. For visual comparison to Figure 1, and discussion of our apoLp-III simulations in the section below, a map for the helix-stabilizing AMBER-94 force field is included (Figure 3a). In our previous work [7], we stated that of the many AMBER force fields studied at that time, “the best agreement with the Protein Data Bank and quantum mechanical sampling is achieved by the AMBER-99φ variant, which captures distributions that are underweighted by other force fields without overweighting other regions of the phase space.” We thus compare both AMBER-99SB and AMBER-03 to our original data set (Figure 3b).

While exhibiting an energetic minimum in the appropriate helical region of the φ/ψ map, the AMBER-99SB variant (Figure 3c) shows a strong bias toward extended β and polyproline conformations (φ<0° and ψ>90°) that is qualitatively similar to that observed for the AMBER-03 potential. As demonstrated in Figure 1, both of these potentials inherently include local minima in this region, with AMBER-99SB including several local minima that span all high ψ values. In addition, AMBER-99SB heavily overweighs the prevalence of left-handed helices in the (φ, ψ) = (57°, 47°) region of the map. Together, these characteristics of AMBER-99SB act to overwhelmingly minimize the helical content of these model helix-forming peptides.

It is certainly of import and interest that our observations regarding the AMBER-99SB force field clearly contradict recent reports that apply this force field to polyalanine peptides and larger proteins, such as the simulational study of ubiquitin by Showalter and Brüschweiler [32], and suggests that AMBER-99SB outperforms its predecessor, AMBER-99, in accurately predicting N-H NMR *S^2^* order parameters. Their limited sampling, however, on the 20 ns timescale for ubiquitin, which is composed of predominantly beta structure with only a small helical region, is not indicative of the ability of this force field to adequately stabilize helical structure on longer timescales. In addition, recent reports by Best and Hummer have shown that the AMBER-99SB force field performs poorly in reproducing experimental helical content for small peptides [33], [34]. Contradicting this report, a recent study by Simmerling and co-workers, developers of the AMBER-99SB force field, reported that this potential performs among the best of currently available models in reproducing experimentally observed *J*-coupling constants [35]. While this concise review of recent studies on force field behavior only scratches the surface of the heavy recent activity within the literature, it is clear that contradictions abound, and we can only report here our observations of the behavior of this system with respect to the well characterized helical F_s_ peptide.

In contrast to the observed behavior of AMBER-99SB, the φ/ψ map representing the AMBER-03 force field shown in Figure 3d is qualitatively similar to that derived using AMBER-99φ. However, some differences are readily apparent. Most importantly, the energetic minimum corresponding to left-handed helix conformations is slightly less favored in the AMBER-03 force field, while the polyproline region of the map is significantly more favorable than observed for the AMBER-99φ potential. In addition, AMBER-03 overweighs the region of the map that corresponds to extended β conformations and, in general, samples a much broader portion of the Ramachandran map than any of the other force fields studied. The lack of proper helical stability for F_s_, as well as the decreased occurrence of 3_10_ helix and mean number of helical segments per peptide (consistently 22% less than observed in AMBER-99φ), are thus attributed to these aspects of the AMBER-03 potential.

To be fair, we must acknowledge recent studies that have made arguments against the use of the AMBER-99φ force field in similar fashion to criticisms of AMBER-99SB [33], [36]. The recent increase in force field assessment has included a number of approaches, a variety of molecular systems ranging from short polyalanine peptides (generally ALA_3_ to ALA_5_) to larger protein systems, and various inconsistencies in methodology, such as the equilibration method followed and the solvation model used. We stress that our analysis of the AMBER-99φ force field, and the comparisons made to other force fields, follows a previously outlined goal [7] of comparing a model *helical* system, the F_s_ peptide, to known experimental observables on a quantitative basis. We have previously shown that this force field does well at reproducing experimental thermodynamic and kinetic quantities for F_s_ [7], but made no claims about the application of this potential to the highly varying chemical moieties upon which the many recent reports described above have been based.

Indeed, following our initial publication of the AMBER-99φ force field, we also reported on the inability of this potential and other AMBER potentials to adequately characterize polyproline type II structure in the blocked ALA_7_ peptide [24], suggesting in that report that force field behavior should be dependent on peptide length, as highly diverging structural character has been observed in varying lengths of polyalanine peptides. While AMBER-99φ was shown to best predict the experimental radius of gyration and alanine *J*-coupling constant for this small polyalanine peptide compared to the AMBER-94, AMBER-GS, AMBER-96, AMBER-99, and GROMOS 53A6 force fields, a lack of experimental evidence for significant helix formation in that peptide suggests that this success was ambiguous. We thus look forward to a more systematic and consistent approach being adopted by the biosimulation community in assessing specific force fields with respect to specific peptide and protein sequences and lengths, and move forward in an effort to assess these force fields with respect to a much larger helical system: the flexible apolipophorin-III helical bundle protein.

### Apolipophorin-III helical bundle protein

**Figure 4:**
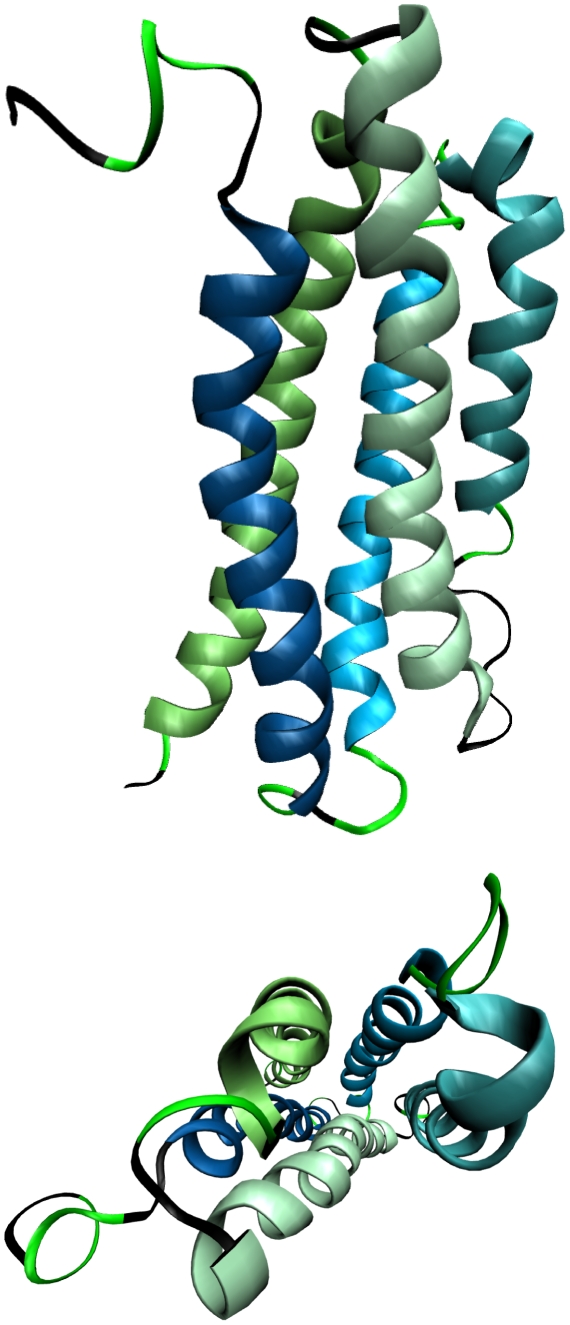
Ribbon views of the NMR model of apolipophorin-III. NMR model 1 of this 164-residue, five-helix-bundle protein (PDB 1LS4) was used to start our simulations in the noted AMBER force fields. Bright green and black unstructured regions represent turn and random coil regions, respectively. Helices are colored from blue (helix 1) to green (helix 5). The bottom view is rotated toward the reader to provide an axial view down the helical bundle central core region.

The NMR structure of the apoLp-III lipid-transport protein used to initiate our simulations is shown in Figure 4, with turn regions colored in bright green and helices 1 thru 5 colored in shades ranging from blue to green, respectively. This protein participates in lipid transport via an apparent “unhinging” or “bundle opening” mechanism [12], which exposes hydrophobic residues within the core of the native fold for lipid binding. It is known to have a relatively low structural stability of ∼2 kcal/mol when folded and a midpoint temperature of T_1/2_∼325 K [37]. Assuming similar folding kinetics to its relative, apolipoprotein A-I, this protein should fold on the seconds timescale [38]. We therefore expect the protein to be structurally stable, but also flexible, at 300 K on the 100 ns timescale. While the data from these 100 ns runs cannot be used to predict thermodynamic stability on longer timescales, they are adequate for assessing the ability of these force fields to model such systems on timescales currently accessible *in silico*.

**Figure 5:**
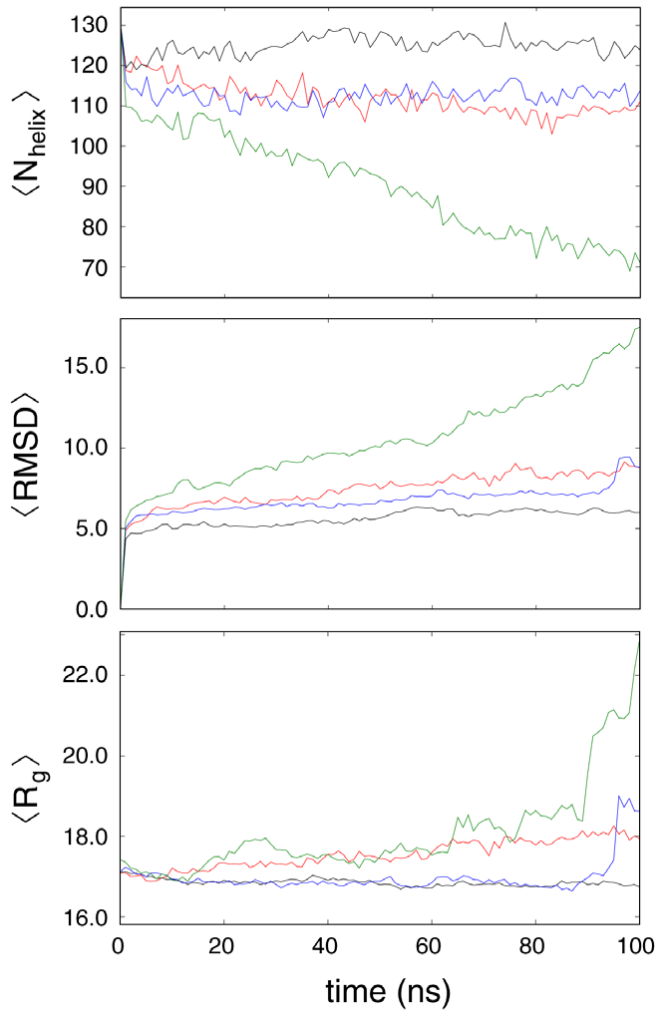
Mean structural properties of simulated apolipophorin-III ensembles. These profiles represent averages over eight 100 ns simulations using the AMBER-94 (black), AMBER-03 (red), AMBER-99φ (blue), and AMBER-99SB (green) force fields. From top to bottom are the average number of helical residues, average all-atom root-mean-square deviation, and average radius of gyration.

Figure 5 shows the time-averaged structural properties of the protein when using the force fields studied herein. For comparative purposes, the seminal Cornell force field, AMBER-94 [3], was also employed in this study. The AMBER-94 force field, while being quantitatively the closest to our AMBER-99φ potential in accurately reproducing experimental values for the F_s_ peptide in our previous study [7], was also shown to significantly overstabilize helical structure and thus serves as a good benchmark by which the helicity of more recently reported potentials can be considered. We note that the AMBER-94 force field maintains the most helical and rigid of structures seen in our 100 ns trials, as would be expected based on our previous results and similar observations reported in the literature.

In contrast, the AMBER-03 and AMBER-99φ potentials both maintain slightly larger RMSD values and slightly lower helicity on average, as was observed for the helical peptides studied herein. The AMBER-03 ensemble does show a slight continued increase in both RMSD and R_g_ over this window, and it is thus not possible to draw conclusions about the behavior of this force field on longer time scales. The AMBER-99φ ensemble shows a more constant behavior across the 100 ns trial window with respect to these structural metrics, with one exception: late in this simulation window a single apoLp-III simulation has the protein undergoing an unhinging motion of helix 1 in which the protein bundle opens, resulting in larger RMSD and R_g_ values, with no significant change in helicity. Motion of this sort has been suggested as a possible means by which lipid moieties are bound for transport [12], and it is thus assumed that such motion is not an indication of loss of structure, but rather a native dynamics of this protein. Further study of this behavior, particularly on much longer time scales, is currently underway.

As is apparent in Figure 5, the lack of helical content observed for small helical peptides under the AMBER-99SB force field was again observed in our simulations of apoLp-III. Unfolding of the protein occurs on the tens of nanoseconds timescale, with the entire ensemble of eight simulations showing distinct unfolding behavior throughout the 100 ns simulation window and the mean number of helical residues decreasing ∼40% alongside a mean RMSD reaching ∼20 Å. This unfolding is also clearly demonstrated in Table 3, which details the mean solvent-accessible surface areas observed in our apoLp-III simulations and amongst the 21 NMR models. The values in Table 3 were generated only after discarding the first 10.0 ns of simulation time in each force field to allow for structural relaxation. Unlike the other AMBER force fields studied, the AMBER-99SB potential shows very large divergence toward greater polar and, much more notably, non-polar surface areas. These large values correspond to the unfolding described in Figure 5.

**Table 3:** Mean SASA* for apolipophorin-III simulations.

Force Field	SASA	SASA_polar_	SASA_nonpolar_
AMBER-94	9163±322	4439±175	4725±285
AMBER-99φ	9309±470	4504±197	4806±418
AMBER-03	9934±698	4777±311	5157±482
AMBER-99SB	10407±1480	4887±606	5521±945
NMR‡ (1LS4)	9051±157	4515±103	4535±113

*SASA (solvent-accessible surface area) is in Å^2^ and was calculated using VEGA (http://nova.colombo58.unimi.it).

‡ All 21 NMR models were used to generate these means and standard deviations.

**Figure 6:**
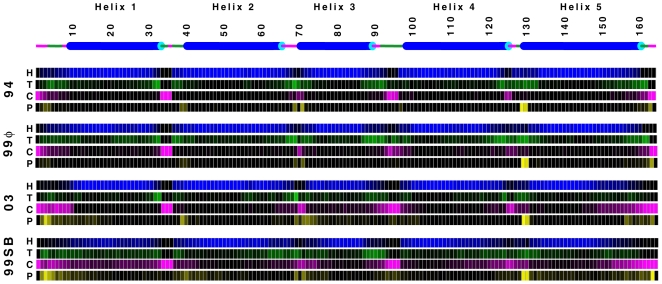
Structural sampling of apolipophorin-III per residue. Probabilities of sampling helix (H), turn (T), random coil (C), and polyproline type II (P) states when simulated using the AMBER-94, AMBER-99φ, AMBER-03, and AMBER-99SB force fields are shown. The schematic at the top represents the NMR model that was used to initiate all simulations, with turns shown in green and coil regions shown in pink to match the color coded state sampling plots below, which show probability ranges from 0.0 (black) to 1.0 (color).

Corresponding structural probabilities per residue are shown in Figure 6 for each of these force fields. The helix schematic at the top of that figure depicts the NMR model used to initiate our simulations, including turn and coil regions. Figure 6 demonstrates a significant extension of helical regions in apoLp-III when employing AMBER-94, primarily into regions that are expected to act as turns between helices. In contrast, AMBER-99φ yields the most flexible molecular shape and size with standard deviations in RMSD and R_g_ of 2.8 and 1.5 Å, respectively, while also preserving a proper molecular size. In comparison, the AMBER-03 potential yields the highest RMSD and R_g_ of the three *helix-friendly* force fields. AMBER-99SB shows a much lower helical content over this timescale than these *helix-friendly* force fields, along with substantial preference for turn and coil conformations. Interestingly, the AMBER-03 and AMBER-99SB force fields show similar polyproline type II conformational content, which is lacking from the AMBER-94 and AMBER-99φ potentials.

We note that AMBER-99φ and AMBER-03 both yield significantly lower mean helical content than AMBER-94, and slightly larger mean RMSD values. While the NMR models provide a picture of the *average* structure of apoLp-III, it is expected that helical regions will fluctuate on the nanosecond timescale, as observed for smaller helical peptides. Moreover, as this protein is expected to undergo some large scale fluctuations to function as a lipid transport protein [12], the observed flexibility of this protein is not unexpected as it is a primary aspect of what makes the apolipoprotein family interesting from both biochemical and computational vantages. Indeed, accurate simulations of these proteins should not maintain the overstabilized helical propensities exhibited in AMBER-94 simulations, nor a tendency to extend helical regions. Both AMBER-99φ and AMBER-03 exhibit significantly less helical propensity in regions that are initially in turn or coil conformations and show global flexibility that should be expected for such proteins.

As noted above, Table 3 lists the mean total SASA, polar SASA, and non-polar SASA predicted by each force field. While it should be noted that the three *helix-friendly* force fields are all in agreement with the NMR ensemble within the errors reported in Table 3, the mean values observed for each force field do show significant differences on the timescale of our study. The NMR models yield a consistently lower total SASA than observed in any of our simulated ensembles. In agreement with the largest RMSD and R_g_, AMBER-03 predicts a total SASA that is ∼10% larger than NMR models, with most of this discrepancy resulting from the exposure of non-polar groups to solvent during simulation. With a total SASA that is closest to the NMR average, AMBER-94 sampling demonstrates an unexpected trend: a decrease in polar SASA below the NMR average and an increase in non-polar SASA well above the NMR average. We postulate that this strikingly counterintuitive behavior results from the extension of helices into turn and coil regions as described above, which would require the rearrangement of sidechains away from their NMR positions, and we are currently investigating this behavior in order to offer a more definitive description of this phenomenon. Of the three force fields, AMBER-99φ yields a reasonably low total SASA and a polar SASA that is nearly identical to the NMR average. As is the case for both AMBER-94 and AMBER-03, the AMBER-99φ potential also exposes significant non-polar SASA to solvent, suggesting a force field independent, systematic trend in all-atom simulations of this protein, which is also currently under investigation.

Finally, the AMBER-99φ and AMBER-03 sampling shown in Figure 6 demonstrates distinct structural trends that should be noted. First and foremost, while these two models do not suffer from the overstabilization of helical structure observed in AMBER-94 simulations, each exhibits clear trends with regard to the turn and coil regions connecting helices 1 thru 5. In our AMBER-03 sampling this includes a significantly larger probability of being in polyproline type II and random coil conformations between helices, with greatly diminished likelihood of turn regions. In stark contrast, AMBER-99φ favors more compact turn conformations over coil and polyproline configurations, thereby explaining the accurate R_g_ and more accurate mean SASA values observed in AMBER-99φ sampling.

*Of these three models which is “best” for simulating large helical proteins?* As is the case in other contexts, proteins that are inherently flexible cannot be well characterized by a single quantitative metric, such as RMSD. We thus address this question by considering other quantitative metrics of structural integrity. While three of these four potentials prove to stabilize apoLp-III simulations, some significant disparities are observed. AMBER-94 overstabilizes helical regions, extending them into the turn and coil linker regions between helices, and strays from the expected trend in moving toward higher polar SASA and lower non-polar SASA. AMBER-03 yields a very reasonable total helical content, yet also overestimates the molecular size, significantly overestimates both components of the SASA, and favors coil and polyproline conformations between helices where turns should be prevalent. Of the three, AMBER-99φ maintains proper molecular size, overall helical content, and polar SASA, while favoring turns in regions between helices rather than coil or extended configurations.

### Conclusions

We have employed several molecular mechanical potentials and evaluated these models to assess their relative accuracy in simulations of helical peptides and proteins. The AMBER-99SB and AMBER-03 potentials were compared to the AMBER-99φ helix-coil force field as applied to polyalanine-based helical peptides and several observations were made. Most notably, the AMBER-99SB potential is decidedly *helix-destabilizing*, as demonstrated by the rapid unfolding of both small helical peptides and the apoLp-III helix bundle on the tens of nanoseconds timescale. We also found that the AMBER-03 potential, while yielding reasonable and qualitatively similar results for these helical peptides when compared to our AMBER-99φ data, also suffers from multiple shortcomings. These include a tendency to show a significant *decrease* in helical content with ALA→ARG^+^ substitutions, a lack of expected 3_10_ helical content, and an overweighing of the extended β and polyproline portions of the Ramachandran map.

These force fields were also employed in simulations of the 164-residue five-helix-bundle apolipophorin-III protein, as was the seminal AMBER-94 force field of Cornell *et al*. This latter potential strongly overstabilized the helical content in apoLp-III, showing a tendency to expand helical regions into the turn and coil linkers separating helices 1 thru 5, while maintaining a very rigid gyration radius of 16.9±0.2 Å, very close to the NMR average of 17.2 Å. Surprisingly, the AMBER-94 data showed distinct shifting to lower polar SASA and higher non-polar SASA, a trend that warrants further investigation. AMBER-03 yielded the largest mean RMSD, R_g_, and SASA values of the three *helix-friendly* potentials, demonstrating a definitive preference for extended and polyproline conformations, as was observed in our equilibrium sampling of small helical peptides.

Of the four force fields discussed above, AMBER-99φ yields the best quantitative results in simulating helical peptides. This includes the occurrence of a low but appreciable 3_10_ helical content, as well as consistent results for the insoluble polyalanine 21-mer and its ARG^+^ substituted analog. In addition, this potential yields nearly perfect agreement with the apoLp-III NMR average gyration radius and polar SASA, while also exhibiting flexibility and fluctuation in helical regions expected of a helix bundle protein with low stability.

In assessing our apoLp-III data, a trend toward increasing non-polar SASA was observed with all AMBER variants tested, the most significant of which (AMBER-03) showed a nearly 14% increase in mean non-polar solvent exposed surface area within 100 ns. There is no doubt that simple point-charge molecular mechanical models, such as the AMBER force fields employed in this study, suffer from imperfections that vary from model to model. We find it striking that these three AMBER variants, which differ significantly both in terms of their respective point-charges and their torsional parameterizations, would consistently favor an increasing non-polar surface area while showing such dramatically different trends in polar SASA. We are thus investigating this phenomenon and look forward to providing a follow-up report on the cause for such trends.
